# Researching and Practicing Positive Psychology in Second/Foreign Language Learning and Teaching: The Past, Current Status and Future Directions

**DOI:** 10.3389/fpsyg.2021.731721

**Published:** 2021-08-19

**Authors:** Yongliang Wang, Ali Derakhshan, Lawrence Jun Zhang

**Affiliations:** ^1^Center for Second Language Writing Research/School of College English Teaching and Research, Henan University, Kaifeng, China; ^2^Department of English Language and Literature, Faculty of Humanities and Social Sciences, Golestan University, Gorgan, Iran; ^3^Faculty of Education and Social Work, The University of Auckland, Auckland, New Zealand

**Keywords:** broaden-and-build theory, positive emotions, positive institutions, positive individual features, positive psychology, second/foreign language education

## Abstract

In addressing the recent special issue in *Frontiers in Psychology*, namely “*Positive Psychology in Foreign and Second Language Education: Approaches and Applications*,” calling language education researchers around the globe to study positive emotions, positive personality traits, and positive institutional tendencies and their implications for language education systems, stakeholders, and policy practices, the present conceptual review paper aims to acquaint language education researchers, practitioners, instructors, and learners with the main tenets of positive psychology and their application in second/foreign language (L2) education research. Accordingly, by drawing on the broaden-and-build theory of positive emotions, we explain how individuals' positivity can result in their flourishment and development in any aspect of life, including L2 learning and teaching. Then, we introduce and conceptualize seven instances of positive psychology variables, namely academic engagement, emotion regulation, enjoyment, grit, loving pedagogy, resilience, and well-being and explain how these positive factors contribute to desirable L2 learning and teaching experiences. Subsequently, potential theoretical and pedagogical implications are drawn to enhance the quality and effectiveness of language education systems and their respective stakeholders. In the end, the limitations of the studies in this area are explicated, and suggestions for future research are provided to expand the extant literature on positive psychology in the domain of L2 education.

## Introduction

In addressing the recent special issue in Frontiers in Psychology, namely “*Positive Psychology in Foreign and Second Language Education: Approaches and Applications*,” the present conceptual review paper aims to acquaint language education researchers, practitioners, instructors, and learners with the main tenets of positive psychology and their application in second/foreign language (L2) education research. This review is different from the work of Dewaele et al. (2019a), entitled “*The Flowering of Positive Psychology in Foreign Language Teaching and Acquisition Research*” in that, in the current review, after introducing the origins, tenets, and theoretical contributions of positive psychology in L2 education, we introduce some key factors which are in need of further empirical investigations and evidence in light of the positive psychology perspective. This review is also different from the work of Dewaele and Li (2020), entitled “*Emotions in Second Language Acquisition: A Critical Review and Research Agenda*” as they have mainly focused on emotions, their different phases, underlying theories, methodological issues, and an agenda for emotions in L2 education.

For years, the domain of educational research was obsessed with investigating negative emotions such as emotions such as anxiety (Marcos-Llinás and Garau, 2009) and burnout (Vaezi and Fallah, 2011) in the instructional context. Discontented with this ever-present focus on negative emotions, researchers inspired by the positive psychology movement have come to realize that not all is negative, and they have attempted to study and encourage eudemonic well-being (Jin et al., 2021; Proietti Ergün and Dewaele, 2021). Positive psychology was first introduced in the seminal work of Seligman and Seligman and Csikszentmihalyi (2000), aiming to advocate a shift in the focus of psychology from a mere concern with fixing the negative and problematic things in life (Gao et al., 2020) to developing positive qualities (MacIntyre, 2021). A few years later, Peterson (2006) defined positive psychology as “the scientific study of what goes right in life, from birth to death and at all stops in between” (p. 4). Seligman and Csikszentmihalyi (2014) have similarly asserted that psychology should shift its focus from identifying and solving problems to the subjective experiences, such as love, hope, and contentment, valued by individuals.

With great attention to individuals' well-being (Mercer, 2021), positive psychology confronts human problems and difficulties from the perspective of strengths rather than weaknesses (Jin et al., 2021). Simply put, while examining the distressing facets of individuals' life were the stock in the trade of psychology for decades, positive psychology has engaged in investigating the positive emotions, strengths, and elements in human experience and psyche (Oxford, 2016). Positive psychology searches for the best in each individual, is concerned with strengths in each person, and fosters individuals' flourishing and well-being (Lopez, 2008). Accordingly, positive psychology is concerned with not only individuals' happiness but also development, fulfillment, flourishment, and resilience in any aspect of life including education (Seligman, 2011).

As identified by Seligman and Csikszentmihalyi (2014), positive psychology is based on the pillars of positive institutions, positive personality characteristics, and positive experiences including emotions. Among these three, positive institutions have been the least investigated one as research in the domain of psychology has mainly attended to positive character strengths and emotions (MacIntyre, 2016). Based on these pillars of positive psychology, researchers in this area study emotions at the levels of (1) the individual (i.e., positive personality characteristics or traits), (2) the subjective (i.e., positive emotional experiences), and (3) the group (i.e., positive support provided by institutions and society) (Gabryś-Barker, 2021). These positive subjective feelings and personality factors, including but not limited to empathy, enjoyment, happiness, contentment, optimism, tolerance, flow, love, and mindfulness, can result in a person's satisfaction, self-efficacy, and success (Seligman, 2011; Fathi et al., 2021).

Positive psychology is theoretically underpinned by the *broaden-and-build theory of positive emotions* which accentuates that positive emotions such as love, interest, joy, and contentment broaden one's “momentary thought-action repertoire” (Fredrickson, 2004, p. 1367). In contrast to negative emotions, which spark the narrowed mindsets, positive emotions foster the broadened mindsets and discovery of creative and new ideas, which can lead to the establishment of one's physical, psychological, intellectual, and social resources (Fredrickson, 2004). Based on Snyder and Lopez's (2009) work, MacIntyre (2016) presents a partial list of 36 potential factors in positive psychology, namely attachment security, benefit-finding, mindfulness, life longings, optimistic explanatory style, personal control, positive growth, self-verification, happiness, resilience, humility, positive ethics, social support, wisdom, relationship connections, meaning in life, self-efficacy, toughness, subjective well-being, gratitude, sustainable happiness, self-determination, character strengths, curiosity and interest, hope, emotional intelligence, optimism, forgiveness, courage, compassion, love, positive emotions, self-esteem, emotional creativity, reality negotiation, and attachment security.

Now that the origin, main tenets, and main foci of positive psychology are touched upon, it is time to see the potential applications of positive psychology in L2 learning and teaching research and practice.

## Positive Psychology in SLA

The prominence of positive emotions and affectivity in L2 learning and teaching was emphasized by early researchers like Arnold (1999); Arnold and Fonseca (2007), and Arnold and Fonseca Mora (2011). However, it was not until rather recently that, following the emergence and rapid flowering of positive psychology in general education, an explicit positive renaissance happened in the domain of language education (MacIntyre and Gregersen, 2012; Lake, 2013). This change urged language researchers and practitioners worldwide to switch their focus from studying negative emotions such as anxiety (Marcos-Llinás and Garau, 2009), boredom (Pawlak et al., 2020), and burnout (Vaezi and Fallah, 2011), to the investigation of both negative and positive factors involved in the process of L2 teaching and learning (Chaffee et al., 2014; Dewaele and MacIntyre, 2014; Kruk, 2019, 2021). In contrast to *the broaden-and-build theory* which disapproves negative emotions and separates them from their positive counterparts, applied positive psychology in L2 education doubts the credibility of the negative-positive polarity of emotions and asserts that negative and positive emotions cannot be easily segregated, and, in many occurrences, they even complement each other (MacIntyre and Gregersen, 2012). Positive emotions can make L2 instruction and learning more enjoyable and personally meaningful and aid L2 instructors and learners to be more resilient in the face of various challenges in the instructional context (Gregersen, 2013).

The special issue on positive psychology guest edited by MacIntyre and Mercer (2014) as well as the pioneering conference at the University of Graz in 2014 on Psychology of Language Learning by Sarah Mercer made the foundations of positive psychology in L2 education more solid. In this respect, the emergence of foreign language enjoyment (Dewaele and MacIntyre, 2014) paved the way for applying PP in SLA and later other positive factors were addressed in this line of research. Accordingly, factors like happiness, emotional intelligence, love, and pride were studied along with negative factors such as foreign language classroom anxiety and negative learning environment in the empirical studies that followed to realize the power of positive emotions for creating a balance (Chaffee et al., 2014; Dewaele and MacIntyre, 2014; Gregersen et al., 2014).

The flowering of positive psychology in L2 education gained rapid momentum in 2016 by the seminal edited books of MacIntyre et al. (2016) and Gabryś-Barker and Gałajda (2016) as well as the second conference on positive psychology in SLA at the University of Jyväskylä. Since 2016, the burst of research papers, adopting a positive psychology perspective in L2 education and being published in more prestigious journals of applied linguistics, has been witnessed (Dewaele et al., 2019a). Works of Mercer (2016); Oxford (2016); MacIntyre et al. (2019), among others, theoretically contributed to this domain.

Among the many contributions that positive psychology has made over the last 20 years, MacIntyre (2016) specifies four of them having direct application in L2 education. The first one is the move from negative to positive emotion, which suggests that emotion will be a promising topic in L2 education, and future research in this domain will greatly benefit from the empirical and theoretical uniqueness of negative and positive emotions and their role in L2 teachers or students' educational outcomes (Li et al., 2020). The second main contribution of positive psychology with potential applicability in L2 education is the model of character strengths (Park et al., 2004), presenting a summary of strengths and virtues under six overarching categories of justice, transcendence, humanity, temperance, courage, and wisdom, which are needed for personal development. When applied in L2 education, this model presents how L2 teachers and students can prosper and flourish in the instructional context by increasing their character strengths (MacIntyre, 2021).

The third contribution pertains to the advancement from PERMA to EMPATICS for understanding well-being within positive psychology (Oxford, 2016). The PERMA model, introduced by Seligman (2011), is a multifaceted concept standing respectively for Positive emotions, Engagement, Relationships, Meaning in life, and Accomplishment. Based on this model, to find connection and meaning in life, there should be an interaction of positivity among all these elements, as a result of which the individual's well-being will be actualized (Mercer and Gregersen, 2020). Later, after reviewing the extant theoretical literature in the area, Oxford (2016) expanded this model and renamed it as EMPATHICS, including the nine components of (1) Emotion and empathy, (2) Meaning and motivation, (3) Perseverance, including hope, resilience, and optimism, (4) Agency and autonomy, (5) Time, (6) Hardiness and habits of mind, (7) Intelligences, (8) Character strengths, and (9) Self factors (self-verification, self-esteem, self-concept, and self-efficacy). Based on this theoretical model, new empirical and pedagogical vistas are opened for SLA researchers. As argued by Oxford (2016), many of the factors in EMPATHICS, such as character strengths, hope, empathy, and resilience have not been addressed in EFL/ESL language teaching and learning, which means that language education research can benefit from attention to this important factors by theoretically drawing on positive psychology.

The last contribution to L2 education research discussed in this paper is the concept of flow, which is indeed one of the founding concepts in positive psychology. According to the flow theory (Csikszentmihalyi, 1990), flow is a state of positive well-being happening when individuals are functioning at the edge of their capacities, where abilities and challenges work together harmoniously, establishing a feeling that one is so engrossed in the task at hand that he/she loses track of time. Although little research has been done on flow in the domain of SLA (Liu and Song, 2021), it is a promising avenue for research in the future as L2 learners' experience of flow can directly influence their L2 learning attainment and success.

Having explicated the prominence of positive psychology in the domain of L2 education, in the next section, we introduce and review seven instances of positive psychology factors, which are in need of dire attention by researchers in the domain of L2 learning and teaching research.

## Positive Psychology Factors

### Enjoyment

MacIntyre and Gregersen (2012) introduced the concept of foreign language enjoyment (FLE), arguing that enjoyment, as an instance of positive achievement emotion (Pekrun, 2006), can help learners build resources for better language learning, broaden their perspectives, and increase their engagement in the language learning process (Jin and Zhang, 2019). It is conceived as a desirable experience happening when learners feel capable of successfully completing the task at hand (i.e., the control element) and appreciating the learning content (i.e., the positive appraisal element) (Mierzwa, 2019). Enjoyment stimulates sustainment in action, which leads to flourishment and development in life. Through building on positive emotions, language teachers can concurrently decrease foreign language anxiety and increase foreign language enjoyment in their learners (Dewaele et al., 2018; Dewaele and Dewaele, 2020). Research studies on FLE have focused on its conceptualization, measurement, antecedents, and correlates in the language learning process (e.g., Li et al., 2018; Jin and Zhang, 2019). Reviewing the existing literature indicated that FLE can lead to better academic achievement (Jin and Zhang, 2018; Li et al., 2020), L2 motivation (MacIntyre, 2016), and social-behavioral learning engagement (Dewaele and Li, 2021).

Research evidence on the contributing factors to FLE has rather unanimously indicated that teacher-related factors play a more important role than learner-related factors in FLE. In this respect, teacher variables such as emotional support, use of humor, level of friendliness, respect toward students, tone of voice, and positive mood were found to influence learners' FLE (Dewaele et al., 2019c). Thus, teachers play a significant role in building enjoyment in the foreign language education ecology. From a methodological perspective, many studies on FLE were mixed methods approaches where participants' voices were heard in descriptions of episodes they enjoyed in their FL classroom or in subsequent interviews (e.g., Dewaele and MacIntyre, 2014, 2019; Li et al., 2018; Li, 2020).

In 2019, Mierzwa asserted that FLE is “an underestimated and not fully explored emotion” (Mierzwa, 2019, p. 173). However, it should be noted that recently, many studies on FLE have been conducted from different perspectives including scale development, expansion of FLE nomological network, individual focus, and longitudinal orientation (Elahi Shirvan and Taherian, 2018; Elahi Shirvan and Talebzadeh, 2018, 2020; Jin and Zhang, 2019; Elahi Shirvan et al., 2020, 2021; Talebzadeh et al., 2020). Thus, it is no longer an underestimated emotion. Nevertheless, the following points can be recommended as future directions of FLE research: expansion of the range of time frames; shifting form simple correlational designs toward more complex statistical techniques capturing the dynamic interactive variables; and further exploratory and experimental research in classrooms.

### Well-Being

Another main factor within positive psychology is well-being. Oxford (2016) accentuated its importance by stating that “positive psychology is all about human well-being” (p. 21). In general, terms, well-being refers to individuals' satisfaction with their life, physical and mental health, and work (Garg et al., 2014). As a positive health-related outcome, well-being promotes individuals' growth and flourishment (Seligman and Csikszentmihalyi, 2000). Ryff (1989) conceptualized well-being as encompassing seven main elements of positive relations with others, self-acceptance, personal growth, purpose in life, environmental mastery, and autonomy. According to Seligman's (2011) PERMA model, well-being is conceived as happening from the interaction of positivity in relationships, accomplishment, meaning, positive emotions, and engagement with the ultimate aim of finding meaning in life (Mercer and Gregersen, 2020). Within the domain of L2 education, understanding and promoting teachers' and students' well-being, whether at its emotional or psychological level, is very important since it lies at the heart of language teaching and learning (Mercer, 2021). Well-being can bring about positive emotional and academic experiences for both teachers and learners such as students' higher FLE (Proietti Ergün and Dewaele, 2021) and teachers' more work engagement and better emotion-regulation (Greenier et al., 2021). It should be noted that compared to teacher well-being, student well-being is a less investigated topic. Thus, it is a promising avenue for future studies in domain-specific research areas like L2 learning.

### Resilience

As a latent intrapersonal positive quality with great contribution to general and language education domains, resilience refers to a dynamic, modifiable developmental process happening overtime reflecting the capacity to adjust to different circumstances or situations and enhance one's effectiveness when confronted with unfavorable conditions (Bobek, 2002). The necessity of resilience for teachers' and students' effective functioning is very noticeable as instruction and learning are stressful and emotionally draining processes (Gu and Day, 2013). In the instructional context, resilience can be defined as one's employment of all resources at his/her disposal to sustain his/her well-being and productivity in the face of daily hardships (Gu and Day, 2013). Through resilience, teachers and students can confront the difficulties and stressors that they face in the instructional context and, rather than just surviving, they are empowered to flourish at institutes, universities, or schools (Gloria et al., 2013). Language teaching profession is essentially relationship-based, where teacher resilience can be developed in the interactions of the teacher with students, and as a result, teachers can find purpose and meaning in their profession and engage in meaningful actions (Hiver, 2018). Resilience has been associated with different L2 educational outcomes such as learning motivation (Kim and Kim, 2021), well-being, and teaching enjoyment (Proietti Ergün and Dewaele, 2021). Despite its eminence, resilience is in dire need of attention to its measurement, contributors, and potential consequences as there is a narrow body of research evidence on this concept in the language education literature (Hiver, 2018).

### Emotion Regulation

Another variable within positive psychology that helps L2 teachers and students to function more effectively in the classroom ecology is emotion regulation (Greenier et al., 2021). Emotion regulation pertains to different behavioral, physiological, or cognitive processes that one uses to regulate emotional experiences and expressions (Gross and John, 2003). Thompson (2008) defines emotion regulation as extrinsic and intrinsic processes that an individual goes through to evaluate, modify, or control his/her emotions to accomplish specific purposes and goals in life. Gross (1998) considers emotion regulation as an interpersonal undertaking, relating to one's ability to regulate when and how one should express and experience emotions. In the instructional domain, emotion regulation strategies are recurrently employed by effective teachers and students. Sometimes, they may down-regulate negative emotions to hinder undesirable influences on performance while at some other times they may up-regulate positive emotions to enhance their efficacy, create an enjoyable learning environment, and more adroitly handle educational tasks (Teng and Zhang, 2018; Greenier et al., 2021).

Teachers or students' knowing how to skillfully regulate both positive and negative emotions in the language classroom contributes a lot to effective teacher-student interpersonal relationships, students' learning gains, and teachers' successful teaching (Ghanizadeh and Moafian, 2010; Teng and Zhang, 2016). Regulating emotions enable teachers to skillfully face conflict situations and find practical solutions for them (Ghanizadeh and Moafian, 2010). Despite its importance for educational outcomes and quality, research evidence on emotion regulation in language education is scanty (Bielak and Mystkowska-Wiertelak, 2020). It is also conceived that Dewaele and Dewaele (2020) while emotions are the lynchpins to language education, they have been mainly ignored in SLA research. Emotion regulation has been studied in relation to such L2 academic factors as psychological well-being, engagement (Greenier et al., 2021), self-efficacy (Fathi and Derakhshan, 2019), reflection (Fathi et al., 2021), and reduced burnout (Ghanizadeh and Royaei, 2015). It is hoped that following the recent flowering of positive psychology in L2 education which highlights studying individuals more holistically by understanding the role of both positive and negative emotions in their development (Gabryś-Barker and Gałajda, 2016), more studies on how L2 teachers or students up-regulate and down-regulate their positive and negative emotions, respectively, be conducted in the near future.

### Academic Engagement

One of the desirable students' experiences in the language learning domain is their academic engagement (Jiang and Zhang, 2021; Khajavy, 2021), which is conceptualized as encompassing the emotional, cognitive, and behavioral dimensions (Reschly and Christenson, 2012). Behavioral engagement refers to learners' actual inclination to participate in tasks and lessons (Mercer, 2019). Emotional engagement is conceived as students' feelings of devotion and attachment to a task. Cognitive engagement happens when one is adequately, mentally challenged, and absorbed in one's work (Reschly and Christenson, 2012). Compared to other well-established and extensively researched constructs like motivation, engagement is regarded as a fledging concept (Hiver et al., 2021) as Reschly and Christenson (2012) also call it “a new kid on the block” (p. 4). Nevertheless, despite its somewhat short history, engagement has gained outstanding popularity in educational research (Mystkowska-Wiertelak, 2020).

The recent seminal works of Hiver and other scholars in SLA have contributed a lot to the extension of student engagement into the L2 education domain. Hiver et al. (2021) conceptualized L2 engagement as the degree that a language learner is mentally or physically involved in doing a language learning task. Mercer and Dörnyei (2020) asserted that language learners' willing engagement is a desideratum for developing communicative language ability, which requires extensive communicative practice and involvement. Many language education stakeholders believe that a key factor to increasing L2 students' ultimate attainment and success is fostering their engagement (Mercer, 2019). To precipitate the expansion of research in this domain, Hiver et al. (2020b) designed and piloted survey instruments to measure language learners' level of engagement in the language learning context across the behavioral, emotional, and cognitive dimensions. But, it needs to be mentioned that in comparison with the domain of educational research, language learning is at its infancy, having just initiated to study L2 engagement in connection with learning different languages in different contexts and cultures (Hiver et al., 2020a). Within the extant literature, students' L2 engagement was found to be positively related to teacher care, rapport (Derakhshan et al., 2021a), nonverbal immediacy, and credibility behaviors (Derakhshan, 2021), emotions, grit (Khajavy, 2021), enjoyment (Dewaele and Li, 2021), willingness to communicate (Mystkowska-Wiertelak, 2021), and bilingual/biliteracy learning (Zhang et al., 2012).

### Grit

Grit is defined by Duckworth et al. (2007) as persistence, passion, and effort for long-run goals. They regarded grit as a high-order factor encompassing the two dimensions of consistency of interest and perseverance of effort. The former dimension refers to maintaining interest in an activity even in the face of failures and hardships, while the latter pertains to the inclination toward working hard and making effort even when confronting obstacles and challenges. Grit is positively related to the growth mindset, which can positively predict academic achievement (Steinmayr et al., 2018). An outstanding feature of grit is its malleability, which means that it can be enhanced through intervention and instruction in the instructional context (Clark and Malecki, 2019). In the language classroom, by taking advantage of this malleability of grit, L2 teachers can prepare learners for the potential hardships and challenges of learning a new language. Grit has been found to be positively associated with academic outcomes such as achievement (Akos and Kretchmar, 2017), enjoyment, and willingness to communicate (Teimouri et al., 2020). While this non-cognitive concept has recently grabbed the attention of language education researchers (e.g., Khajavy et al., 2020), the number of studies in this domain is still scanty and more investigations are demanded to expand our understanding of the role of this factor in language learning, which is a prolonged process replete with trial and errors, challenges, and difficulties.

### Loving Pedagogy

Love is one of the desirable emotions propounded by positive psychology, having a great potential to promote humans' flourishment, development, and fulfillment (Fredrickson, 2004; Seligman, 2011). More particularly, in the educational context, love is conceived as a meaningful positive learning experience. Loreman (2011) believes that love plays a prominent role in learners' emotional and social development. Some famous figures like John Lock also assert that successful instruction only happens within a loving context. Similarly, Freire pinpointed that learning and teaching are acts of love (Yin et al., 2019). Maslow's (1954) hierarchy of needs also reflects the prominence of love: individuals can reach self-actualization only when their belonging and love needs are fulfilled. The concept of loving pedagogy mainly reflects the concern with satisfying students' needs. For doing so, teachers need to be caring, supportive, and sensitive regarding their students as well as respecting and understanding them. A loving instructor has this ability to strengthen students' functioning and potentials Love can also potentially create effective coping mechanisms, motivate learners, and be the driving force in language learning (Pavelescu and Petrić, 2018).

A loving pedagogical approach views the goal of education as something beyond the mere transference of knowledge. Such pedagogy unites students with their instructors to jointly search for knowledge and urge students to surpass all their limitations (Yin et al., 2019). Loreman (2011) conceptualized a “pedagogy of love” model, including the main nine elements of kindness, sacrifice, acceptance, bonding, forgiveness, empathy, community, passion, and intimacy. Despite its importance, loving pedagogy has been an under-researched topic in both general and language education domains. To the best of our knowledge, the first and only attempt to extend the concept of loving pedagogy to the realm of language education research has been undertaken in a large-scale, multinational study, which attempted to revalidate the Dispositions toward Loving Pedagogy (DTLP) Scale in the L2 education context and examine its relationship with creativity and work engagement of EFL/ESL instructors (Derakhshan et al., 2021b). Following the importance of love in language pedagogy, L2 researchers are encouraged to instantly shift their attention to this fledgling domain of research.

**Figure 1:**
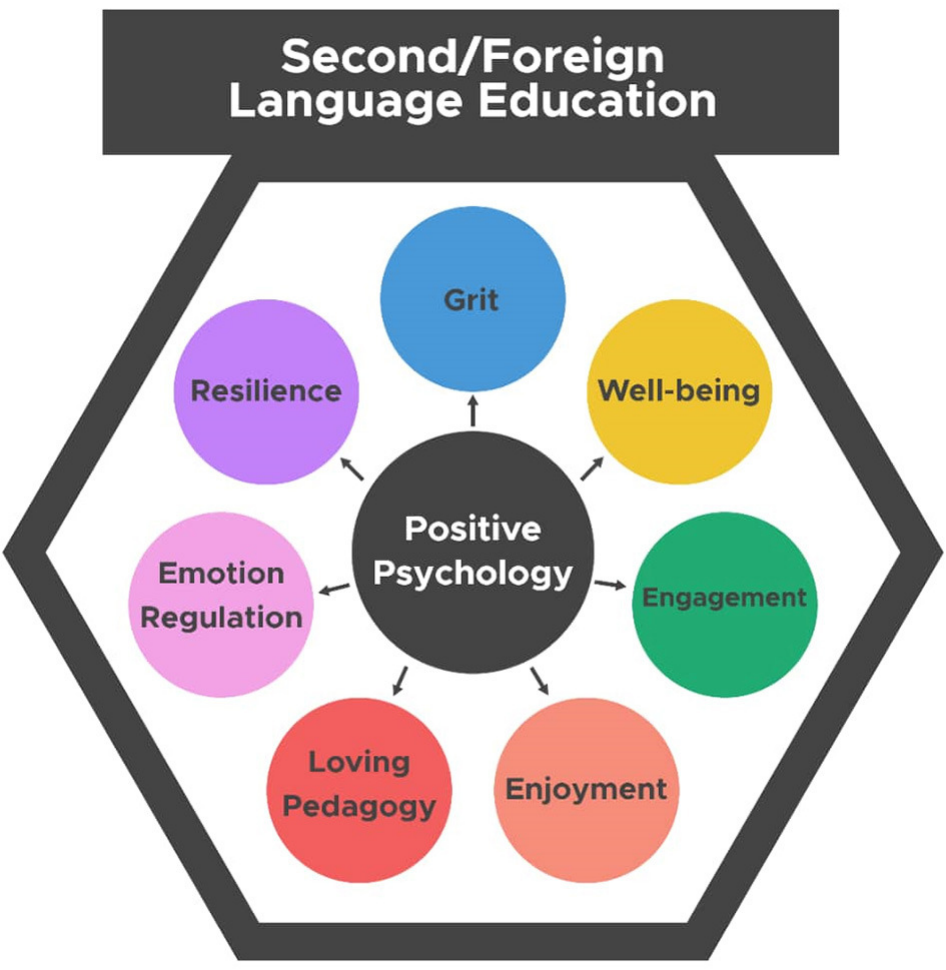
Potential positive psychology factors contributing to the second/foreign language learning experience.

Overall, Figure 1 indicates the visual representation of the positive teacher- or student-related factors discussed so far and their potential applicability in the L2 education research domain.

## Conclusion and Pedagogical Implications

In this review paper, we sketched (1) positive psychology and its main tenets, (2) the theory (i.e., broaden-and-build theory of positive emotions) behind it, (3) the significance of applying positive psychology in L2 education research and practice, and (4) seven instances of positive psychology variables (i.e., academic engagement, enjoyment, grit, emotion regulation, loving pedagogy, resilience, and well-being) and their contributions to L2 student- or teacher-related educational outcomes. In this section, we will show how positive psychology pedagogically contributes to L2 education as it potentially enlightens the functioning of different stakeholders in this domain.

To start with, to date, there has been a neglect to ESL/EFL teachers' well-being concerning their emotional and personal investment into their professional performance. Effective language teaching is not just a matter of conveying the content and pedagogical knowledge and using all the novel teaching methods and techniques (Pishghadam et al., 2019) but also is an emotionally-charged undertaking where language instructors interact and communicate with each learner as they enthusiastically teach the subject matter in an enjoyable learning environment (Xie and Derakhshan, 2021). Thus, the present paper can serve language teacher educators and trainers as they can integrate positive psychology and its positive factors in the teacher education programs, workshops, and courses that they run for enthusiastic pre- and in-service language teachers. In such programs, instruction in positive psychology can help trainee teachers develop their theoretical and practical knowledge by becoming informed of the movement of positive psychology, its main tenets, its prominence in L2 learning and teaching, and the potential teacher or student positive variables contributing to desirable academic outcomes (Gabryś-Barker, 2021).

Consequently, teacher educators should equip language teachers with theoretical and practical knowledge of various positive variables in positive psychology, such as FLE, resilience, and positive emotions and how to apply them in the instructional context with the ultimate aim of increasing teachers' professional effectiveness and enhancing students' L2 attainment and success. As indicated by previous research, teachers play a significant role in preparing a learning environment that can increase students' academic accomplishments (Dewaele and Dewaele, 2020). Accordingly, pre- and in-service teachers should be taught how to create an enjoyable learning environment by showing respect and care toward students, building on positive teacher-student interpersonal relationships (Pishghadam et al., 2021a), and increasing interest in the target language, which will ultimately aid learners sustain engagement with the L2 learning.

Similarly, teacher recruiting committees should broaden the scope of their standards for selecting quality teachers by considering not just teachers' sufficient content and pedagogical knowledge, but also teachers' engagement with their continuing professional development needs (Derakhshan et al., 2020; Wang and Derakhshan, 2021), awareness of new theories and movements in educational research (e.g., positive psychology and broaden-and build theory) and how to apply such theories and research findings in their instructional practice. For instance, the committees can ask the teacher applicants about the strategies that they will employ to increase FLE in their language learners. As shown in previous research, one useful way to aid emergence of FLE in the classroom is teachers' conveying of support and friendliness to students (Mercer and Dörnyei, 2020). Therefore, the authorities in charge of recruiting effective language teachers can target those teachers who are aware of the significance of positive teacher-student relationships, positive emotional experiences, and positive environmental factors in students' enjoyment, effective performance, and success.

## Limitations and Directions for Future Research

It should be acknowledged that there are some limitations in the studies conducted in the domain of positive psychology in L2 education as revealed in its extant literature. First of all, not all positive educational factors have been equally investigated. While some positive variables such as L2 motivation have been extensively researched worldwide (e.g., MacIntyre, 2016; Kim and Kim, 2021), others like L2 engagement have been under-represented in this domain (Hiver et al., 2020a). Based on positive psychology's argument that all positive experiences, personality traits, and institutional variables contribute to individuals' flourishment in any aspect of life including L2 learning and teaching (Seligman, 2011; MacIntyre, 2021), it is incumbent on language researchers and practitioners to study various positive educational variables across cultures, instructional contexts, teachers' instructional experiences, learners' stages of development, or grade levels to understand how they are similar or dissimilar concerning their effects on teachers or learners' academic outcomes (Pishghadam et al., 2021b). Even future research can concurrently investigate more than one of such variables in a single study to see their main and interaction effects on specific individuals' variables.

Second, empirical studies in this domain have been largely quantitative, typically employing close-ended questionnaires to elicit individuals' perceptions and attitudes regarding the particular variables under investigation (e.g., Derakhshan, 2021; Khajavy, 2021). For instance, despite the few qualitative works employing qualitative data collection instruments (e.g., Pavelescu and Petrić, 2018; Dewaele and Pavelescu, 2021), the dominant instrument used to measure student engagement has been self-report surveys and questionnaires. Nevertheless, due to some limitations inherent to this type of data collection method, such as the possible skewness of data, participant bias, and lack of real-time data, they recommend that educational researchers make use of other methods at their disposal such as observations, expert ratings of engagement, interviews, and real-time sampling methods. Therefore, future researchers are advised to switch their focus from purely quantitative studies using close-ended questionnaires to qualitative or mixed-methods research investigations, which can elicit more real time data, conjointly gather different types of data, and enrich our understanding of the phenomena under focus. Within such approaches, they can employ different instruments like narrative writing, audio journal, observation, interview, open-ended questionnaire, field note, or diary. Moreover, besides conducting large-scale studies gathering data from large samples to increase data-to-population generalizability of findings, more future studies can be done on examining perceptions or experiences of a few cases and gathering rich data from them. Longitudinal studies are also beneficial in this area, showing how individuals' perceptions, experiences, or behaviors change or remain the same across time.

Third, while studies in this area have been conducted in different parts of the world, they have been mainly set within one specific context. Except Dewaele and MacIntyre (2014, 2019) and Dewaele et al.'s (2019b) studies which had international samples, there is less empirical evidence on cross-cultural studies, seeking to unravel how individuals from different cultural contexts conceptualize, perceive, and experience positive emotions and characteristics. This is very important because culture is a pivotal factor affecting how our mindset is shaped and reshaped (Pishghadam et al., 2021a). To fill the identified lacuna in the literature, the authors of the present study and their co-researchers have initiated a new line of cross-cultural studies on positive teacher-, student-, or institution-related variables such as teacher rapport, credibility, care, stroking behaviors (a type of teacher care), work engagement, psychological well-being, students' willingness to attend L2 classes, L2 engagement, loving pedagogy in the Iranian, Polish, British, and Iraqi cultures (e.g., Derakhshan et al., 2021a,b; Greenier et al., 2021; Pishghadam et al., 2021a). Nevertheless, positive psychology issues of other cultural contexts still need to be investigated. Thus, future L2 researchers are encouraged to continue this line of cross-cultural positive psychology research and extend the established theories and areas of research across the globe.

Fourth, advances in technology have affected every aspect of humans' life, and language education is not an exception here. The digital world offers numerous possibilities for language learning and teaching to contemporary language learners and teachers. Online learning provides opportunities for practicing the language with native and non-native speakers from different cultures as well as holding classes in emergency situations, such as the COVID-19 pandemic, which can drastically disrupt the language education provision (Wang and Derakhshan, 2021). It should be noted that positive educational factors rightfully occupy a prominent place in the virtual language education literature. Thus, future researchers are urged to study factors such as resilience, loving pedagogy, well-being, engagement, grit, and enjoyment in both traditional and digital settings and unravel how each of the two learning systems might affect language learning and teaching experiences.

Fifth, little research (Li and Xu, 2019) has been conducted on how the mentioned positive psychology variables can be integrated into L2 education programs with the aim of enhancing the thinking and functioning of key stakeholders, namely language teachers, and students. While many of the previous studies have confirmed the importance of positive variables like FLE, grit, and engagement for predicting desirable academic outcome (e.g., Teimouri et al., 2020), there is a shortage of studies for showing how individuals can be empowered by receiving intervention and instruction on these factors. To fill this research gap, future studies can conduct experimental studies by exposing a group of language learners or instructors to treatment on a specific positive academic variable and see how explicit instruction can enhance their perceptions, experiences, or reactions.

All in all, what this conceptual review paper endeavors to show is that positive psychology in L2 learning and teaching is a vast line of research, which currently is at its initial stage of development. Therefore, this fecund area of research is enthusiastically receptive of language researchers across the globe to do empirical investigations on its under-represented aspects to precipitate the expansion of this line of research.

## Author's Note

We believe that the present conceptual review paper can acquaint language education researchers, practitioners, instructors, and learners with the main tenets of positive psychology and their application in second/foreign language (L2) education research. Accordingly, by drawing on the broaden-and-build theory of positive emotions, we explain how individuals' positivity can result in their flourishment and development in any aspect of life, including L2 learning and teaching. Then, we introduce and conceptualize seven instances of positive psychology variables, namely academic engagement, emotion regulation, enjoyment, grit, loving pedagogy, resilience, and well-being and explain how these positive factors contribute to desirable L2 learning and teaching experiences. Subsequently, potential theoretical and pedagogical implications are drawn to enhance the quality and effectiveness of language education systems and their respective stakeholders. In the end, the limitations of the studies in this area are explicated, and suggestions for future research are provided to expand the extant literature on positive psychology in the domain of L2 education.

## Author Contributions

All authors listed have made a substantial, direct and intellectual contribution to the work, and approved it for publication.

## Conflict of Interest

The authors declare that the research was conducted in the absence of any commercial or financial relationships that could be construed as a potential conflict of interest.

## Publisher's Note

All claims expressed in this article are solely those of the authors and do not necessarily represent those of their affiliated organizations, or those of the publisher, the editors and the reviewers. Any product that may be evaluated in this article, or claim that may be made by its manufacturer, is not guaranteed or endorsed by the publisher.
